# Inhibitory Effects of Amorphigenin on the Mitochondrial Complex I of *Culex pipiens pallens* Coquillett (Diptera: Culicidae)

**DOI:** 10.3390/ijms160819713

**Published:** 2015-08-20

**Authors:** Mingshan Ji, Yaping Liang, Zumin Gu, Xiuwei Li

**Affiliations:** Department of Pesticide Science, College of Plant Protection, Shenyang Agricultural University, Shenyang 110866, China; E-Mails: jimingshan@syau.edu.cn (M.J.); lyp628@syau.edu.cn (Y.L.); guzumin1212@163.com (Z.G.)

**Keywords:** *Culex pipiens pallens*, amorphigenin, mitochondrial complex I, inhibitory mechanism, kinetics

## Abstract

Previous studies in our laboratory found that the extract from seeds of *Amorpha fruticosa* in the Leguminosae family had lethal effects against mosquito larvae, and an insecticidal compound amorphigenin was isolated. In this study, the inhibitory effects of amorphigenin against the mitochondrial complex I of *Culex pipiens pallens* (Diptera: Culicidae) were investigated and compared with that of rotenone. The results showed that amorphigenin and rotenone can decrease the mitochondrial complex I activity both *in vivo* and *in vitro* as the *in vivo* IC_50_ values (the inhibitor concentrations leading to 50% of the enzyme activity lost) were determined to be 2.4329 and 2.5232 μmol/L, respectively, while the *in vitro* IC_50_ values were 2.8592 and 3.1375 μmol/L, respectively. Both amorphigenin and rotenone were shown to be reversible and mixed-I type inhibitors of the mitochondrial complex I of *Cx. pipiens pallens*, indicating that amorphigenin and rotenone inhibited the enzyme activity not only by binding with the free enzyme but also with the enzyme-substrate complex, and the values of *K*_I_ and *K*_IS_ for amorphigenin were determined to be 20.58 and 87.55 μM, respectively, while the values for rotenone were 14.04 and 69.23 μM, respectively.

## 1. Introduction

*Culex pipiens pallens* Coquillett, 1998, is the most common mosquito in houses of Northern China, Korea and Japan, and is the primary vector of filariasis, epidemic encephalitis B [1] and a potential vector of the West Nile virus [2]. Control of *Cx. pipiens pallens* populations in China has been provided principally by the use of various contact and residual insecticides since the 1950s [3]. However, 50 years of sustained struggle against harmful mosquitos using synthetic and oil-derivative molecules has produced pervasive secondary effects such as mammalian toxicity, mosquito population resistance to organochlorine, organophosphate, carbamate and pyrethroid insecticides [4,5,6,7,8], and ecological hazards. Hence, much attention must be taken to develop alternatives to chemical insecticides for mosquito control.

Among the alternative strategies, the use of plants, insecticidal phytochemicals appears to be promising. More than 2000 plant species have been known to produce chemical factors and metabolites of value in pest control programmes [9]. In recent years, many studies on plant extracts and new active molecules to combat mosquito larvae have been conducted around the world, and some novel mosquito larvicidal compounds have been isolated and identified [10]. Those plant materials and isolated compounds are known to possess biological activity such as insecticidal activity, repellency, reproduction retardation, and insect growth regulation for instance against various mosquito species, and have received considerable attention in the search for new biopesticides as potential mosquitocides, as summaried by Kishore *et al.* [11]. Although numerous reports are available concerning the larvicidal potential of the plant secondary metabolites, the mode of action of botanicals insecticides is still uncertain and most of them are under investigation for their insecticidal mechanisms. Among botanicals tested against mosquitos are the following: the essential oils of *Lippia turbinate* and *Lippia polystachya* (Family: Leguminosae) at doses ranging from sublethal to lethal (20, 40 and 80 ppm) modify the temporal pattern of locomotion of *Culex quinquefasciatus* (Diptera: Culicidae) larvae [12]. *Aedes aegypti* (Diptera: Culicidae) organophosphate susceptible and resistant larvae were distinctly affected by lectins WSMoL and cMoL from the seeds of *Moringa oleifera* (Family: Moringaceae). The determination results of digestive (amylase, trypsin, and protease) and detoxifying (superoxide dismutase (SOD), α- and β-esterases) enzymes indicated that the larvicidal mechanism of WSMoL may involve the deregulation of digestive enzymes, while cMoL interfered mainly on SOD activity [13]. Furthermore, the trypsin inhibitor MoFTI from *M. oleifera* flower extract interfered with the survival and development of *A. aegypti* larvae and killed bacteria inhabitant of larvae midgut [14]. Four purified flavones, one flavanone and a diterpenoid isolated from *Andrographis paniculata* Nees (Family: Acanthaceae) exhibited an inhibitory effect on the cytochrome P450 monooxygenases CYP6AA3 and CYP6P7 of *Anopheles minimus* (Diptera: Culicidae) [15]. The crude extract of *Agave sisalana* of the Agavaceae family can cause cell lysis and destruction of the peritrophic membrane, reduce the concentration of NO in the hemolymph from *A. aegypti* larvae [16]. Acetylcholinesterse, β-carboxylesterase and acid phosphatases activity were significantly reduced in *A. aegypti* larvae exposed to the aqueous kernel extract of soapnut *Sapindus emarginatus* belongs to the family Sapindaceae [17]. Two constituents of the Alaskan yellow cedar tree, the monoterpenoid carvacrol and the sesquiterpenoid nootkatone, are both toxic against several arthropods. Carvacrol was observed to cause slight inhibition of the acetylcholinesterase enzyme in house flies, ticks and cockroaches, but it did not inhibit the mosquito acetylcholinesterase enzyme. Nootkatone did not inhibit the acetylcholinesterase enzyme in any of the four arthropod models tested [18]. Thus, mode of action and site of effect for larvicidal phytochemicals and extracts has received little attention [10].

**Figure 1:**
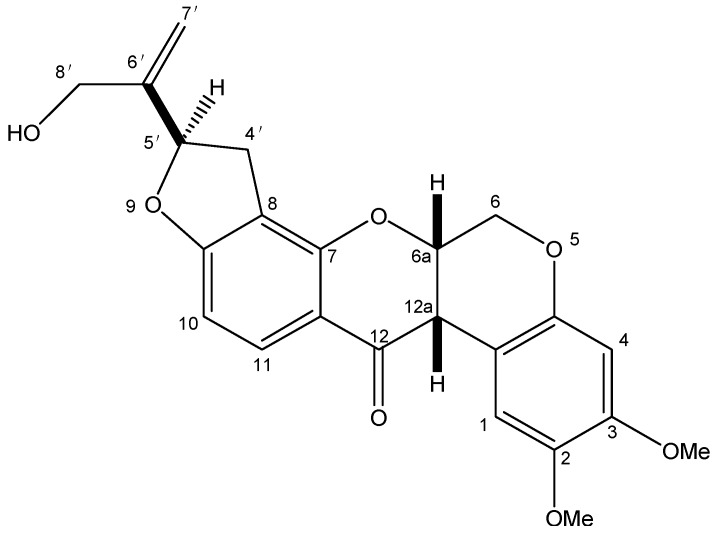
Chemical structure of amorphigenin.

Amorphigenin (Figure 1), an aglycone of the rotenoid glycoside amorphin [19,20], has been isolated from the leaves, seeds and seedlings of *Amorpha fruticosa* [21,22] and has been shown to have significant anti-proliferative [23], anti-cancer (in many cell types) [24,25], hepatoprotective [26] and neuraminidase inhibition [27] activities. As for insecticidal activity, earlier research of the 1940s showed that extracts of *A. fruticosa* possessed repellent and insecticidal properties [28,29], and the acetone extract of *A. fruticosa* seeds to be more toxic against *A. aegypti* larvae than 1% pure rotenone [29]. Also, amorphigenin 8′-β-glucoside at 10 ppm was shown to lead to an 85% loss of the fourth instar larvae of *A. aegypti* and without the formation of pupae [30]. Our previous study [31] showed that ethanol extract from seeds of *A. fruticosa* had good contact effect and antifeedant activity against *Schizaphis graminums* (Homoptera: Aphididae). Then, amorphigenin, a rotenoid compound which exhibits a strong larvicidal activity with LC_50_ and LC_90_ values of 4.29 and 11.27 mg/L, respectively, was isolated from the ethanol extract by column chromatograpy [32]. However, up to now little is known about inhibition effect of amorphigenin on mosquito larvae. Indeed, insecticidal activity and inhibitory effect on mitochondrial respiratory complex I of rotenoids are clearly different. For example, 5′β- epirotenone, the stereoisomer of natural rotenone, was about 1000-times less inhibiting the active of mammalian NADH-ubiquinone reductase than rotenone [33]. The toxicity of 5′β- epirotenone against 3th instar larvae of *Bombyx mori* (Lepidoptera: Bombycidae) was about 24-times less than rotenone, and the LC_50_ values of two compounds were 225.70 and 9.33 mg/L, respectively [34]. In consideration of similar the chemical structure of amorphigenin and rotenone [22], we can not simply deduce whether amorphigenin possess similar inhibition mechanism with rotenone. So bioassay and enzyme kinetics study are needed.

The mitochondrial complex I (NADH-ubiquinone oxidoreductase, EC 1.6.5.3) catalyzes the first step of oxidative phosphorylation and, hence, it is the key for the efficient ATP production in most prokaryotic and eukaryotic cells [35]. The enzyme transfers electrons from NADH to ubiquinone which is the terminal electron acceptor. The inhibition of the complex I results in the termination of ATP production. It is well known that the mitochondrial complex I can be specifically inhibited by natural products such as piericidin, rotenone and rotenoids [36], and annonaceous acetogenins [37]. Photoaffinity labeling research showed that arylazidoamorphigenin can inhibit the bovine heart mitochondrial complex I activity at concentrations comparable with those of rotenone [38].

Although a large number of complex I inhibitors have already been reported, the inhibitory effect of these were rarely investigated. The purpose of the present work is, therefore, to carry out a bioassay and a kinetic study of the mitochondrial complex I activity inhibition of *Cx. pipiens pallens* mitochondria and to evaluate kinetic parameters and inhibition mechanisms of amorphigenin. In addition, these data can provide the basis for the development of novel and effective mosquito larvae control agents.

## 2. Results and Discussion

### 2.1. Results

#### 2.1.1. *In Vivo* and *in Vitro* Inhibition of the Mitochondrial Complex I Activity in *Cx. pipiens pallens*

The *in vivo* inhibitions of the mitochondrial complex I activities by amorphigenin and rotenone were compared among the fourth-instar larvae of *Cx. pipiens pallens* exposed to water containing the solvent alcohol (control) and different concentrations of amorphigenin and rotenone for 24 h. Amorphigenin at 1.25, 2.5 and 5 μmol/L decreased the mitochondrial complex I activities by 1.52-, 1.78-, and 2.43-fold, respectively, compared with those of the control when NADH was used as the substrate (Table 1). Similarly, rotenone at 1.25, 2.5 and 5 μmol/L decreased the mitochondrial complex I activities by 1.45-, 1.77- and 2.41-fold, respectively.

**Table 1:** The *in vivo* and *in vitro* inhibition of amorphigenin and rotenone against the mitochondrial complex I of *Cx. pipiens pallens*.

Drug Delivery	Compounds	Regression Equation	IC_50_ (μmol/L)	*R* Value
*In vivo*	Amorphigenin	*y* = 4.0495 + 2.4616*x*	2.4329	0.9046
	Rotenone	*y* = 3.9764 + 2.5466*x*	2.5232	0.9152
*In vitro*	Amorphigenin	*y* = 3.0076 + 4.3668*x*	2.8592	0.9884
	Rotenone	*y* = 3.2495 + 3.5250*x*	3.1375	0.9982

Significant inhibitions of the mitochondrial complex I activities by amorphigenin and rotenone were also observed in *in vitro* assays. When NADH was used as the substrate, Amorphigenin at 2 μmol/L decreased the mitochondrial complex I activity by 27.38% as compared with the control whereas amorphigenin at 3 and 4 μmol/L decreased the enzyme activities by 50.00% and 70.24%, respectively. Furthermore, parallel assays using rotenone, a known the mitochondrial complex I inhibitor [39], were performed. The results showed that rotenone at 3 and 4 μmol/L significantly decreased the mitochondrial complex I activities when NADH was used as a substrate. Table 1 shows the IC_50_ values and linear regression equations of amorphigenin and rotenone against the mitochondrial complex I. The *in vivo* IC_50_ values of amorphigenin and rotenone were determined to be 2.4329 and 2.5232 μmol/L, respectively, while the *in vitro* IC_50_ values of amorphigenin and rotenone were 2.8592 and 3.1375 μmol/L, respectively.

#### 2.1.2. The Effect of Amorphigenin and Rotenone on the Mitochondrial Complex I

**Figure 2:**
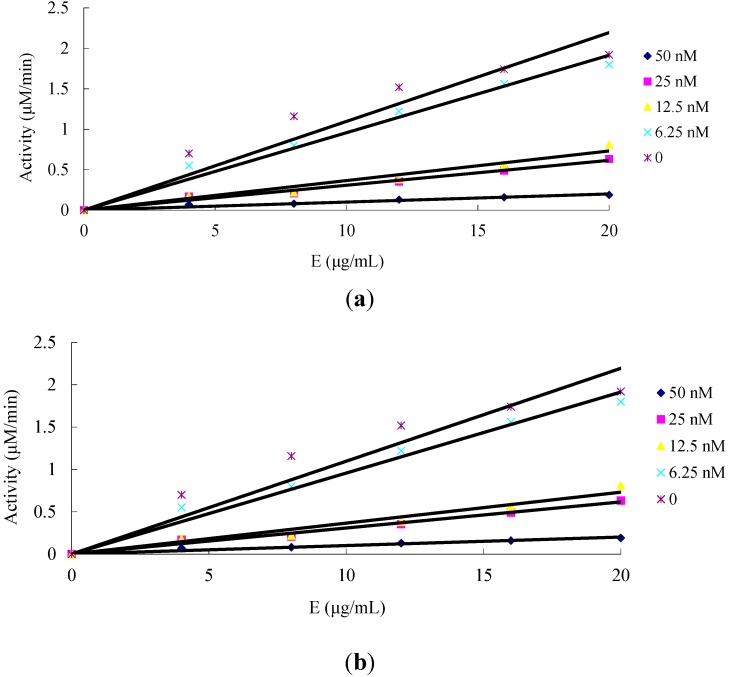
The effect of mitochondrial complex I concentration on catalysis activity of β-Nicotinamide adenine dinucleotide, reduced disodium salt hydrate (NADH) at different concentrations of amorphigenin (**a**) and rotenone (**b**).

The inhibition effect on the mitochondrial complex I by amorphigenin and rotenone was also studied. Figure 2 shows the relationship between enzyme activity and enzyme concentration in the presence of different concentrations of amorphigenin and rotenone. The plots yielded straight lines passing through the origin. Increasing the inhibitor concentration resulted in decreasing the slope of the lines, thus the presence of amorphigenin and rotenone did not decrease the amount of effective enzyme, but simply inhibited and decreased the enzyme activity, indicating that inhibition of amorphigenin and rotenone on the mitochondrial complex I was reversible.

#### 2.1.3. Inhibition Kinetics of Amorphigenin and Rotenone on the Mitochondrial Complex I Activity

**Figure 3:**
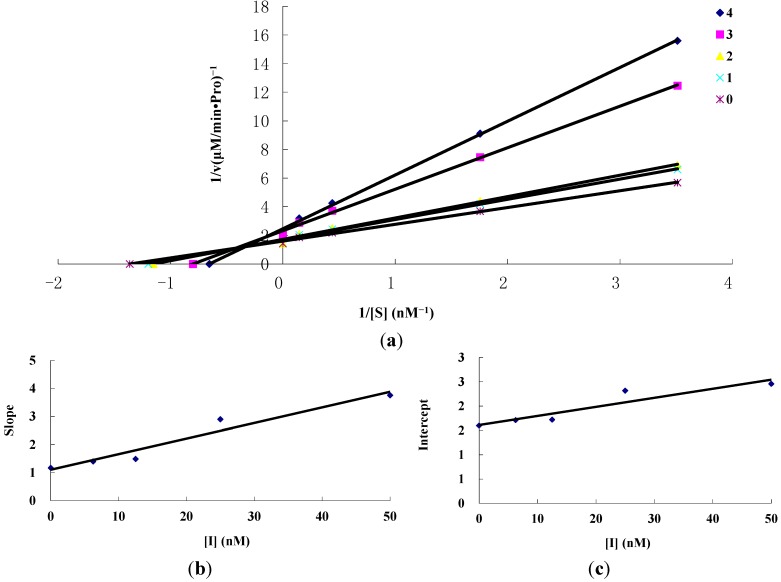
Inhibition kinetics of amorphigenin on mitochondrial complex I by Lineweaver–Burk plots. (**a**) Concentrations for curves 0–4 were 0, 6.25, 12.5, 25 and 50 nM, respectively; and (**b**,**c**) represent the secondary plots of the slope an d intercept of straight line *versus* concentration of inhibitor, respectively.

The inhibitory kinetics of the mitochondrial complex I by amorphigenin and rotenone were studied by Lineweaver–Burk plots. Figure 3 shows the double-reciprocal plots of the enzyme inhibited when using amorphigenin as the inhibitor. Under the experimental conditions employed, the oxidative reaction of NADH by the mitochondrial complex I followed Michaelis–Menten kinetics. The double-reciprocal plots yielded a family of straight lines with different slopes and intercepts but they intersected one another in the second quadrant. Thus, it was a mixed type inhibitor, indicating that amorphigenin inhibited the enzyme activity not only by binding with the free enzyme but also with the enzyme-substrate complex. Equilibrium constants for inhibitor binding with the free enzyme (*K_I_*) and with the enzyme-substrate complex (*K_IS_*) can be calculated according to the Foumulations (1) and (2) based on the slope and the vertical intercept against the concentration of inhibitors in Figure 3, respectively [40].

(1) $$Slope=\frac{K_{s}}{V_{m}}\left(1+\frac{\left[I\right]}{K_{I}}\right)$$

(2) $$\;Intercept=\frac{1}{V_{m}}\left(1+\frac{\left[I\right]}{K_{IS}}\right)$$

**Figure 4:**
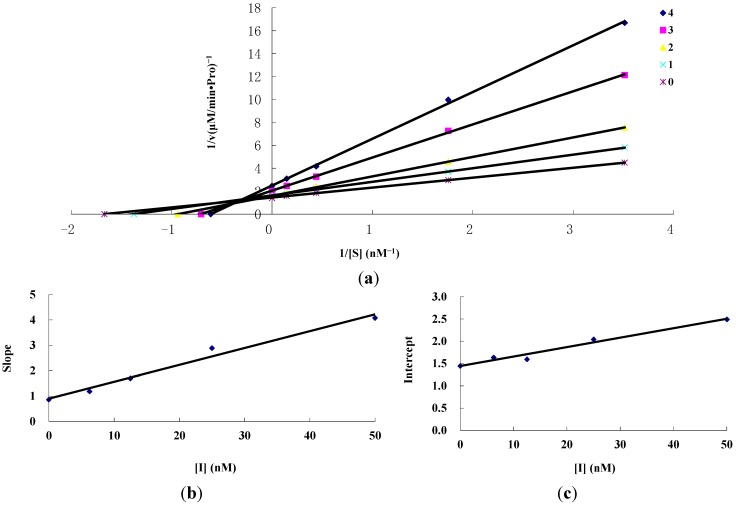
Inhibition kinetics of rotenone on mitochondrial complex I by Lineweaver–Burk plots. Concentrations of (**a**) for curves 0–4 were 0, 6.25, 12.5, 25 and 50 nM, respectively; Figure (**b**) and (**c**) represent the secondary plot of the slope and the intercept of the straight lines *versus* concentration of inhibitor, respectively.

From Figure 3b,c, the values of *K*_I_ and *K*_IS_ for amorphigenin were determined to be 20.58 and 87.55 μM, respectively. Figure 4 shows the results for rotenone, where the inhibition behavior was found to be the same as amorphigenin. The values of *K*_I_ and *K*_IS_ for rotenone were determined to be 14.04 and 69.23 μM, respectively. The value of *K*_IS_ was approximately four times as high as *K*_I_, indicating that the affinity of the inhibitor for the free enzyme was stronger than that for the enzyme–substrate complex.

### 2.2. Discussion

The mitochondrial complex I catalyzes the transfer of two electrons from NADH to ubiquinone, coupled to the translocation of four protons across the inner mitochondrial membrane. The generated electrochemical proton gradient drives energy-consuming processes such as ATP synthesis [41]. The mitochondrial complex I can be specifically inhibited by many natural products [36]. It has been reported that rotenone and rotenone reduced with NaBH_4_ were the most potent inhibitors of the mitochondrial complex I prepared from a pig’s heart [39] with *in vitro* IC_50_ values of 1.8 and 2.0 × 10^−5^ μmol/mg protein, respectively. An *in vitro* study by Earley *et al.* showed that amorphigenin and arylazidoamorphigenin were potent inhibitors of the mitochondrial complex I, comparable with rotenone [38].

**Figure 5:**
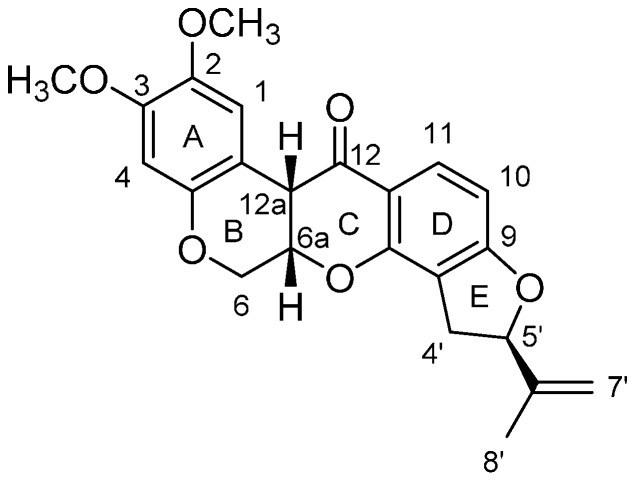
Structure of natural rotenone.

Our study showed inhibition of the mitochondrial complex I of *Cx. pipiens pallens* larvae both *in vivo* and *in vitro* in the presence of amorphigenin and rotenone. These results showed that amorphigenin is a potent inhibitor of the mitochondrial complex I of *Cx. pipiens pallens* larvae with similar IC_50_ values to rotenone. The IC_50_ values obtained from the *in vivo* and *in vitro* assays can be attributed to similar chemical structures in the two compounds. Natural rotenone (Figure 5) consists of a five-ring structure (A- to E-rings) and has three chiral centers (6a-C, 12a-C and 5′a-C) [42]. Structure-function relationship studies of rotenone analogues suggest that the A-B cycles of rotenone mimic the quinone ring of ubiquinone [43], the C-D-E cycles must functionally correspond to the hydrophobic isoprenyl tail of ubiquinone [44]. The rotenone-binding site recognizes the whole molecular structure (or shape) of rotenone in a strict sense [45]. Structure-activity study of a structurally systematic set of rotenone analogues showed that the stereochemical configuration of B and C rings is a very important factor for the activity [39], the modification of the E-ring moiety can affect both the inhibitory potency and the pattern of inhibition, and the inhibitory potency increased with an increase of the hydrophobicity of the portion corresponding to the E-ring moiety [33]. Furthermore, the configuration of the isopropenyl group attached to 5′a-C atom of the E-ring is also important for the activity [43], but the π-electron system of the isopropenyl group attached to the E-ring does not contribute to the inhibitory action [33]. However, the substituents in the A-ring results in almost complete retention of activity [43]. Spectroscopy research showed that amorphigenin differed from rotenone by the presence of a hydroxy group in the substituent on the isopropenyl group [22], and further research indicated that modifications to this region of the rotenoid structure did not seem to affect the inhibitory potency of the mitochondrial respiratory chain complex [38]. Thus, the amorphigenin exhibit the same resistance risk as rotenone. Until to now, as an insecticide which has been in use for more than 150 years, only two rotenone resistant strains of *Spodoptera eridania* (Lepidoptera: Noctuidae) [46] and *Leptinotarsa decemlineata* (Coleoptera: Chrysomelidae) [47] were occasionally found in the field. Taking into account the chemical instability, easy degradation of rotenone, which all its toxicity will lost in 2–3 days of summer sunlight [48], the use of rotenone will give little selection pressure on pests. So the potential resistance risk of rotenone [49] and analog amorphigenin was optimistic. However, further investigation is necessary to fully assess the resistance risk.

To investigate the mechanism of the inhibition of the mitochondrial complex I of *Cx. pipiens pallens* larvae by amorphigenin in this article, we used NADH and endogenous ubiquinone as substrates to determine the complex I activity, inhibitory type and inhibition constant. The results indicated that amorphigenin and rotenone were reversible and mixed-I type inhibitors of the mitochondrial complex I of *Cx. pipiens pallens*. This conclusion however, conflicted with the observations of others. Indeed, several studies about the inhibitory mechanism of rotenone have provided contradictory results. The research of Crombie and Charalambous showed that rotenone was a reversible competitive inhibitor of the mitochondrial complex I from submitochondrial particles obtained from blow fly (*Calliphora erythrocephala* (Diptera: Calliphoridae)) flight-muscle [50]. Meanwhile, the research of Ueno [33] indicated that rotenone inhibited NADH-ubiquinone reductase of bovine heart submitochondrial particles in a noncompetitive manner against exogenous quinine DB and DPB. Alternatively, these contradictory results may have been due to the origin of the mitochondria, the membrane bounded enzyme or partially purified complex I substrate, and/or the experimental conditions [51]. In our study, amorphigenin and rotenone inhibited the enzyme activity not only by binding with the free enzyme but also with the enzyme-substrate complex, and the affinity of the inhibitor for the free enzyme was stronger than that for the enzyme-substrate complex.

## 3. Experimental Section

### 3.1. Chemicals

Amorphigenin, derived from the seeds of *Amorpha fruticosa* by the authors, was identified by ^1^H nuclear magnetic resonance. Rotenone was purchased from Guangxi Shile Agrochemical Co., Ltd. (Nanning, China). Bicinchoninic acid disodium salt hydrate (BCA), β-nicotinamide adenine dinucleotide, reduced disodium salt hydrate (NADH) and bovine serum albumin (BSA) were purchased from Sinopharm Chemical Reagent Co. (Shanghai, China). The other chemicals used in this study were of analytical grade. The deionized water used was from a Milli-Q reagent water system (Millipore, Bedford, MA, USA).

### 3.2. Mosquito Culture

*Culex pipiens pallens* was maintained in our laboratory without exposure to any insecticide, at 27 ± 2 °C with 75%–85% relative humidity and a 12:12 L/D photoperiod. The larvae of *Cx. pipiens pallens* were reared in a plastic basin containing a sterilized diet (40 mesh goat liver powder/yeast (2:1) in water). Adult mosquitoes were maintained on a 10% sucrose solution and blood from a mouse.

All procedures performed on animals within this study were conducted following guidelines of the Association for Assessment and Accreditation of Laboratory Animal Care International (AAALAC).

### 3.3. Isolation of Mitochondria

The mitochondria of the fourth instar larvae of *Cx. pipiens pallens* were isolated according to the method of Akbar *et al.* [52] with suitable modifications. The larvae were washed in a cold extraction medium (0.25 M sucrose solution containing 0.25% defatted BSA), then homogenized in a Potter–Elvehjem homogenizer under cold conditions. The homogenate was filtered through a moist muslin cloth and the filtrate centrifuged at 1000× *g* for 15 min at 4 °C. The residue was re-suspended in the extraction medium and centrifuged at 1000× *g* for 15 min at 4 °C. The supernatants from both centrifugations were combined and centrifuged at 12,000× *g* for 15 min at 4 °C. The mitochondrial pellet was re-suspended in the extraction medium, and stored at −80 °C until used. The protein concentration was determined by Smith’s method [53] using BSA as the standard.

### 3.4. Assay of the Mitochondrial Complex I Activity and in Vitro Inhibition

The mitochondrial complex I activity of *Cx. pipiens pallens* was determined as described by Birch-Machin *et al.* [54] with adaptations for a 96-well format. It is well known that the mitochondrial complex I has two substrates, NADH and ubiquinone. Rotenone and its analogue do not compete with ubiquinone [55,56,57], therefore in this study we used NADH and endogenous ubiquinone of isolated mitochondria as substrates to determine the complex I activity. The activity was measured by following the decrease in absorbance due to the oxidation of NADH at 340 nm with 425 nm as the reference wavelength. In brief, 20 μL of mitochondria and 160 μL of assay buffer containing 25 mM phosphate buffer (pH 7.2), 5 mM MgCl_2_, 2 mM NaN_3_ and 2.5 mg/mL BSA were added to each well of the 96-well plate, then the microplate was incubated for 20 min at 37 °C in a microplate reader (Spectramax 190 plate reader; Molecular Devices, Sunnyvale, CA, USA). Finally, the reaction was initiated by the addition of 10 μL of 0.13 mM NADH and measured for 5 min.

For the assay of *in vivo* inhibition of the mitochondrial complex I activities, first, the fourth-instar larvae of *Cx. pipiens pallens* were exposed to sub-lethal concentrations of amorphigenin and rotenone [32] for 24 h, then isolated the mitochondria from the survival tested mosquito larvae and determined the mitochondrial complex I activity as described above. For the assay of *in vitro* inhibition of the mitochondrial complex I activities, 10 μL of different concentrations each of amorphigenin and rotenone were prepared in alcohol and diluted in 25 mM phosphate buffer (pH 7.2), then mixed with 10 μL of the mitochondria. The enzyme activity was immediately determined by the method as described above. For negative controls, 10 μL of 25 mM phosphate buffer (pH 7.2) instead of amorphigenin or rotenone were used in the assays. The inhibitory extent of the compounds was expressed as the inhibitor concentrations leading to 50% of the enzyme activity reduction (IC_50_).

### 3.5. Determination of the Type and Constant of Amorphigenin and Rotenone Inhibition of the Mitochondrial Complex I Activity

Different concentrations of NADH were incubated with the mitochondria and assay buffer to examine the enzyme kinetics of NADH with and without the addition of various concentrations of rotenoids. The inhibition type of the compounds on the enzyme was assayed by Lineweaver-Burk plots. The inhibition constant was determined by plots of the apparent 1/*V*_m_ or *K_m_*/*V_m_ versus* concentration of the inhibitor [40,58].

### 3.6. Statistical Analysis

Applying Microsoft Excel 2003 software (Microsoft, Redmond, WA, USA), the average enzyme activitie results, adjusted by Abbott [59], were subjected to probit analysis to calculate IC_50_ and correlation coefficient.

## 4. Conclusions

Continuous and excessive application of insecticides has resulted in the rapid development of insecticide resistance in several mosquito species, including *Culex pipiens pallens*. Therefore, it is urgent to find alternative compounds to conquer the vector of periodic filariasis and deadly encephalitides. Many extracts and isolated compounds from plant materials are known to possess biological activity such as insecticidal activity, repellency, reproduction retardation, and insect growth regulation for instance against various mosquito species. However, the mode of action of botanicals insecticides is still uncertain and most of the plant secondary metabolites are under investigation for their insecticidal mechanisms. The present study represents a systematic research about inhibition effect, the mode of action of bioactive compounds having mosquito larvicidal activity from the seeds of *A. fruticosa*. This study improves knowledge of the potent effect of amorphigenin on mitochondrial complex I activity of *Cx. pipiens pallens* larvae.

Investigation of inhibitory effect against *Cx. pipiens pallens* demonstrates that amorphigenin and rotenone can decrease the mitochondrial complex I activity both *in vivo* and *in vitro.* Both amorphigenin and rotenone were shown to be reversible and mixed-I type inhibitors of the mitochondrial complex I of *Cx. pipiens pallens.* Our results indicate that amorphigenin is strong candidate for a natural, safe and effective phyto-larvicide to be used in population control of *Cx. pipiens pallens.*
